# Extent and Degree of Shoreline Oiling: *Deepwater Horizon* Oil Spill, Gulf of Mexico, USA

**DOI:** 10.1371/journal.pone.0065087

**Published:** 2013-06-12

**Authors:** Jacqueline Michel, Edward H. Owens, Scott Zengel, Andrew Graham, Zachary Nixon, Teresa Allard, William Holton, P. Doug Reimer, Alain Lamarche, Mark White, Nicolle Rutherford, Carl Childs, Gary Mauseth, Greg Challenger, Elliott Taylor

**Affiliations:** 1 Emergency Response Division, Office of Response and Restoration, National Ocean Service, National Oceanic and Atmospheric Administration, Seattle, Washington, United States of America; 2 Research Planning, Inc., Columbia, South Carolina, United States of America; 3 Owens Coastal Consultants, Ltd., Bainbridge Island, Washington, United States of America; 4 Atkins, Tallahassee, Florida, United States of America; 5 Polaris Applied Sciences, Inc., Kirkland, Washington, United States of America; 6 EML Environmental Mapping Limited, Saanichton, British Columbia, Canada; 7 Triox, Montréal, Quebec, Canada; University of California, Merced, United States of America

## Abstract

The oil from the 2010 *Deepwater Horizon* spill in the Gulf of Mexico was documented by shoreline assessment teams as stranding on 1,773 km of shoreline. Beaches comprised 50.8%, marshes 44.9%, and other shoreline types 4.3% of the oiled shoreline. Shoreline cleanup activities were authorized on 660 km, or 73.3% of oiled beaches and up to 71 km, or 8.9% of oiled marshes and associated habitats. One year after the spill began, oil remained on 847 km; two years later, oil remained on 687 km, though at much lesser degrees of oiling. For example, shorelines characterized as heavily oiled went from a maximum of 360 km, to 22.4 km one year later, and to 6.4 km two years later. Shoreline cleanup has been conducted to meet habitat-specific cleanup endpoints and will continue until all oiled shoreline segments meet endpoints. The entire shoreline cleanup program has been managed under the Shoreline Cleanup Assessment Technique (SCAT) Program, which is a systematic, objective, and inclusive process to collect data on shoreline oiling conditions and support decision making on appropriate cleanup methods and endpoints. It was a particularly valuable and effective process during such a complex spill.

## Introduction

The *Deepwater Horizon* spill released a U.S. Government-estimated 4.9 million barrels of oil into the Gulf of Mexico over an 87-day period, from 20 April to 15 July 2010 [1] [2]. The fate of the oil included direct recovery from the wellhead, containment, offshore skimming, controlled in-situ burning, natural and chemical dispersion (both subsea and on the surface), and other pathways, including stranding on the shoreline.

In anticipation of shoreline oiling, the Unified Command managing the emergency response (lead by the Federal On-Scene Coordinator [U.S. Coast Guard] in consultation with the State On-Scene Coordinators from each State, and BP) established a Shoreline Cleanup Assessment Technique (SCAT) Program on 28 April 2010. The SCAT process is a well-established and internationally recognized component of spill response in use since the *Exxon Valdez* spill, where a standard methodology for documentation, terminology, and decision making for shoreline assessment and treatment was first applied [3]. Once oil strands on shorelines, responders survey the affected areas to determine the appropriate response. There are many general guidelines for how to best remove the oil from different shoreline habitats and specific cleanup recommendations integrate field data on shoreline habitats, type and degree of shoreline oiling, site-specific physical processes, and resources at risk. Every oil spill is a unique combination of conditions that have to be factored into the development of effective treatment guidelines. During the *Deepwater Horizon* response, oil came ashore over an extended period of time, requiring response activities to be spread over four states over multiple years.

The objectives of this paper are to provide information on the maximum extent and degree of shoreline oiling from the *Deepwater Horizon* oil spill as observed and characterized through methodologies applied for response purposes, as well as shoreline oiling conditions at one and two years post-release, and to describe some of the unique factors of this spill as they pertain to how oil stranded and persisted. This type of information is highly pertinent to oil spill response scientists tasked with contingency planning and responding to future incidents.

## Methods

During the *Deepwater Horizon* spill response, up to 18 SCAT teams, consisting of Federal, State, local, and BP representatives, conducted field surveys to document the location, degree, and character of shoreline oiling using standard methods and terminology. As of January 2013, this effort involved over 7,000 SCAT team-days during which 7,058 kilometers (km) of shoreline were surveyed; however, over 31,000 km of total shoreline has been surveyed, because of the many repeated surveys of the same sections of shoreline over time. These data were the basis for developing shoreline treatment recommendations for specific shoreline segments, using cleanup criteria developed through consensus based on habitat type and use. Following shoreline cleanup treatments, SCAT teams inspected each segment against these criteria. Guidelines for cleaning oiled shorelines have been developed through government and industry funded research, lessons learned from previous spill responses, and on-site tests. The Office of Response and Restoration, National Oceanic and Atmospheric Administration has developed general guidelines for cleanup strategies and cleanup endpoints as part of their role as Scientific Support Coordinator to support the U.S. Coast Guard [4] [5] [6]. These guidelines were used as the discussion starting point for the cleanup criteria that were established for the *Deepwater Horizon* response. Guidelines vary based on the oil properties, season, and habitat type and use.

**Figure 1:**
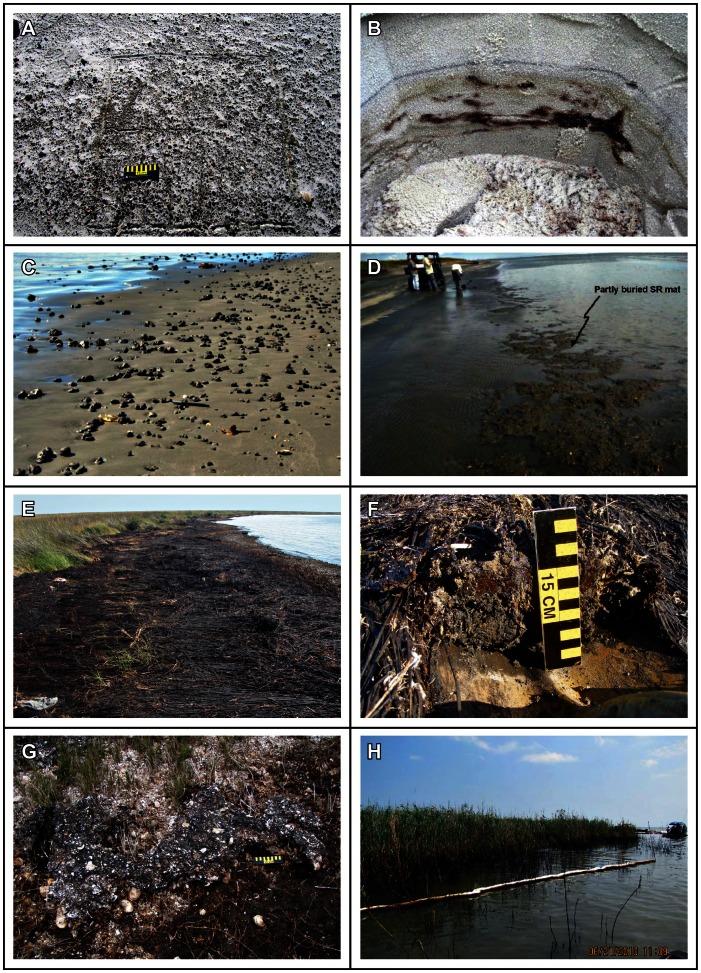
Representative photographs of shoreline oiling conditions. Sand beaches: A. Small surface residue balls in the supratidal zone (scale is 15 cm); B. Buried oil patties; C. Surface residue balls in the intertidal zone that are angular, indicating that they were eroded from adjacent oil residue mats; D. Intertidal oil residue mats at the toe of the beach. Marshes: E. Heavily oiled wrack at the high-water line and oiled mat of laid-over vegetation; F. Thick (>1 cm) emulsified oil under the laid-over vegetation mats; G. Oil/shell incipient asphalt pavement on the marsh platform; H. Oiled *Phragmites* along the Mississippi River delta.

A SCAT survey consists of a team walking the shoreline or transiting close to shore by boat to document oiling conditions using standard terms [4] [7] for oil character, thickness, percent distribution, width and length of the oiled band(s), tidal zone where the oil band(s) were observed, the average and maximum size of oil deposits, and recommended cleanup tactics. The character of the oil that stranded onshore was different than many other spills because the oil was released at the seafloor, rose through approximately 1,500 meters (m) of water, was treated by dispersants both subsea and on the surface, and had to be transported by wind and currents for 80–300 km through warm Gulf of Mexico waters to reach the shoreline. The oil that eventually stranded on the shoreline was in the form of a thick, viscous emulsion, containing up to 60% water, as opposed to fresh, liquid oil. In most cases this emulsified oil stranded as discrete patches, rather than a continuous slick. In marshes, the emulsified oil pooled on the surface with little penetration into the marsh soils. On some sand beaches, the oil penetrated up to a few centimeters (cm) into the sediments, forming a semi-cohesive oil/sediment matrix, referred to as surface oil residue (SR). To reflect the different oiling characteristics observed during the response, SCAT terminology was modified to include surface residue balls (SRBs, <10 cm), surface residue patties (SRPs, >10 cm), and large SR mats that could be 100 s of m long and up to 20 cm thick. Samples of SRBs collected in January 2011 consisted of 4.2–12.8% oil and 87.2–95.8% sand [8]. These SRBs are different from “tarballs” commonly found following oil spills because they are mostly sand and the oil components are not tarry; instead, they are tarball-sized pieces of sand, shell, and other beach materials loosely bound by surface oil residue. Figure 1 shows representative photographs of the types of oil stranded on sand beaches and marshes.

The shoreline response program encompassed four stages, defined primarily to recognize changes in oiling threat, oiling conditions, progression through cleanup operations, and seasonal factors [9] as summarized below:

### Stage I/II Nearshore and Shoreline Response

These stages (May to September 2010) covered the period during which oil continued to strand onshore. Oil spill cleanup tactics create an intrinsic level of environmental impact and the standard approach is to initiate shoreline cleanup once the risk of further shoreline oiling has abated. The ongoing release from the wellhead during this incident required that shoreline cleanup begin while oil was still coming ashore. SCAT shoreline surveys during this Stage were rapid and focused on locating bulk shoreline oiling for immediate response. Shoreline cleanup consisted of removal of floating oil adjacent to the shoreline and bulk oil removal from the shoreline, especially where such oil could remobilize and spread to other areas.

### Stage III Shoreline Response

This stage (September 2010 to March 2011) began once significant quantities of floating oil no longer remained on the sea surface, addressed all shorelines within the Area of Response, and included detailed SCAT surveys. The end of Stage III was a target date to meet cleanup goals by spring 2011, when shoreline use by birds, sea turtles, and people increases. Shoreline Treatment Recommendations (STRs) generated within the SCAT program and approved by the Unified Command were issued for each shoreline segment where treatment was authorized, specifying the area and types of shoreline cleanup operations to be conducted. Acceptable and proven cleanup actions in the affected habitats (sand beaches, marshes, and man-made structures) were identified by groups of representatives from the Responsible Party, Federal, State, and Local jurisdictions to meet cleanup goals building on practices that have evolved during past spills and become encoded into best practices for oil spill response. The goal was to meet the “2010 No Further Treatment (NFT) guidelines” that were developed for each habitat type and to lay the groundwork for future stages of cleanup. NFT guidelines vary from spill to spill, depending upon a variety of factors, such as habitat type and the nature, character, and extent of the oiling. In this instance, the NFT guidelines were developed through consensus by representatives from the Responsible Party and Federal and State jurisdictions. These NFT guidelines were designed to be qualitative and recognizable to both cleanup workers and assessment teams. The objective was to proceed with shoreline treatment until the actions were no longer effective or caused more harm than good and began to slow the recovery process (in other words, proceed until a Net Environmental Benefit was achieved).

### Stage IV Shoreline Response

This stage (March to November 2011, the latter being the end of hurricane season in the United States) consisted of a resurvey of all affected shorelines to document Spring 2011 conditions and determine the need for cleanup to meet “2011 NFT guidelines.” The 2011 NFT guidelines were developed through the same process as the 2010 NFT guidelines. New Stage IV STRs were issued for shorelines requiring treatment based on the oiling conditions documented at the time. Shoreline segments that met the 2011 guidelines were removed from active response. Many segments moved into a patrol and maintenance phase once they met the 2011 NFT guidelines because of the risk of re-oiling from remobilization or re-exposure of subsurface oil on the beaches, as well as oil in nearshore subtidal mats and on marsh platforms.

### Shoreline Cleanup Completion Plan (SCCP) [10]

This final stage of the shoreline response (November 2011 and forward) defined the process whereby removal actions would be deemed complete and shoreline segments could be moved out of the response. For the first time, shoreline-oiling conditions documented by SCAT teams were compared against shoreline cleanup “endpoints,” meaning that once a segment met these final criteria, shoreline treatment was completed. As with the NFT guidelines, the SCCP endpoints were developed through consensus by representatives from the Responsible Party and Federal and State jurisdictions. The Plan included surveys of selected shoreline segments after the 2011 Atlantic hurricane season, and multiple surveys of segments post-treatment to assure that oiling conditions continued to meet endpoints. Segments that did not meet endpoints were returned to Operations for further treatment, and the inspection process was repeated.

SCAT data on oiling characteristics were used routinely to generate maps and tabular data on degree of oiling by habitat over time. Oiling degree categories (Heavy, Moderate, Light, Very Light, Trace) were defined based on the width of oiling bands on the shoreline (as measured perpendicular to the shoreline), the percent cover of oil within the band, and oil thickness using a two-step process (Figure S1 in File S1). In the first step, the width of the oil on the shoreline and the percent cover determine an initial oiling degree category; in the second step, the thickness of the oil determines the final oiling category. For example, a shoreline with a >3 m band of oil with 100% coverage is initially classified as Heavy surface cover; however, if the oil thickness is only a stain or film, the final surface oil category is Light; if the oil thickness is >0.1 cm, the final category is Heavy. The length of the shoreline is not considered in determining the degree or category of surface oiling. For example, along a marsh shoreline with highly variable orientation, there could be hundreds of meters of shoreline with no oiling then a section with tens of meters of Heavy oiling where oil stranded, adjacent to another section with Light oiling. The combination of surface oil categories and lengths of oiled shoreline provide a general level of understanding of the extent and magnitude of a spill; however, these descriptors are not adequate by themselves to develop cleanup strategies and goals for each habitat type or shoreline segment. The selection of appropriate cleanup strategies is dependent upon site-specific information regarding oiling thickness, width, distribution, and character, as well as numerous other factors including habitat condition and sensitivity, public use, wildlife use (e.g. nesting bird colonies, sea turtle nesting), and access and safety concerns.

## Results

**Figure 2:**
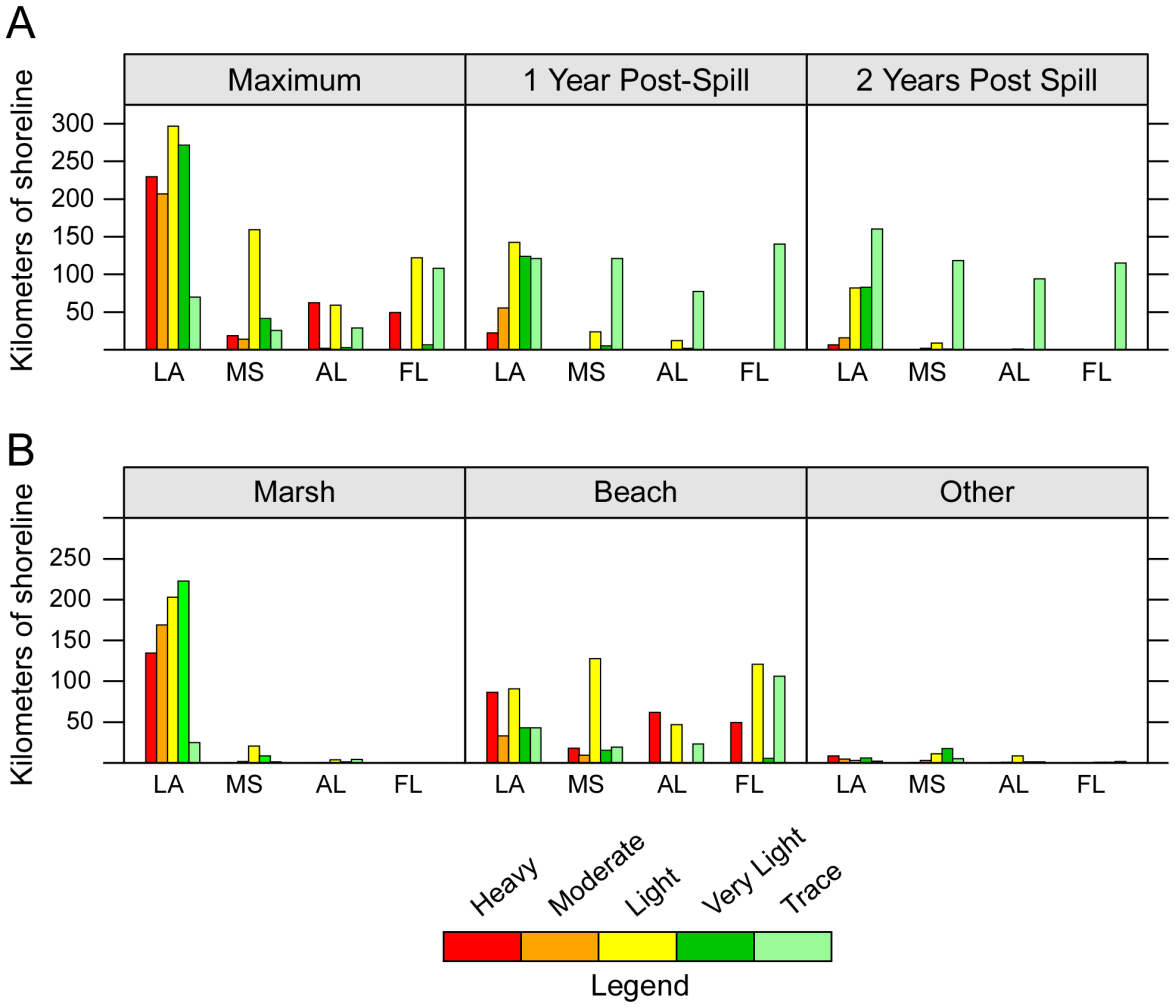
Oiled shoreline lengths (km) by oiling category and State. A. At maximum oiling conditions, one year (May 2011), and two years (May 2012) post spill. B. Oiled shoreline lengths (km) by oiling category, State, and habitat at maximum oiling conditions.

**Figure 3:**
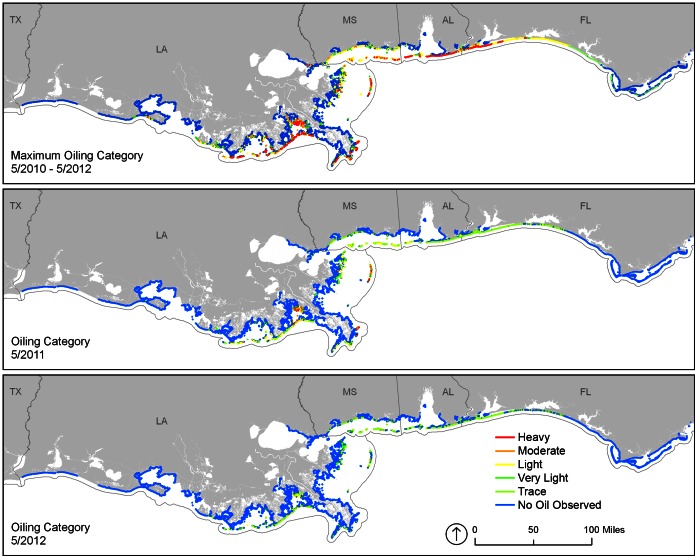
Maps by shoreline oiling category at maximum oiling conditions, one year (May 2011), and two years (May 2012) post spill.

**Figure 4:**
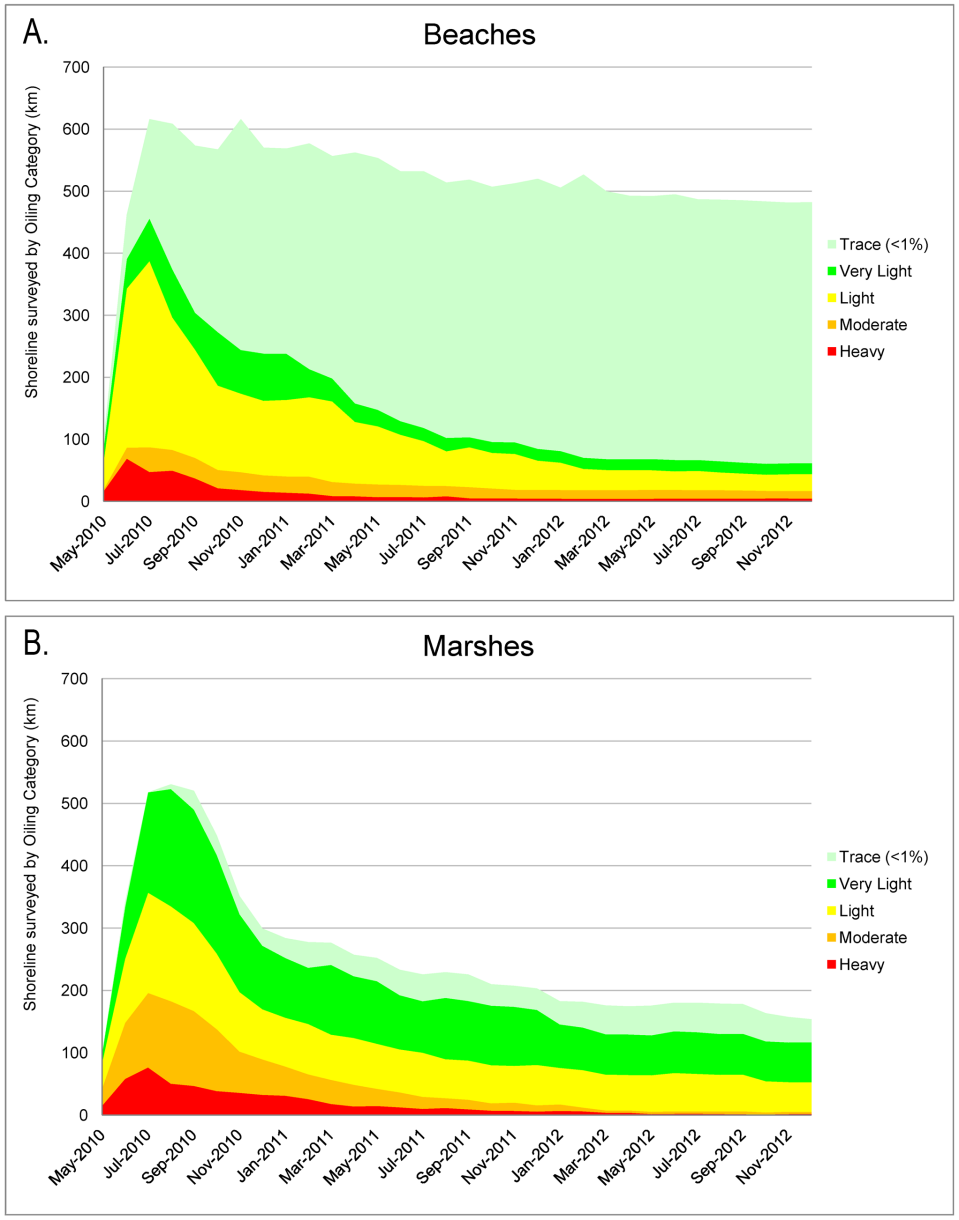
Time-series plots of the km of shoreline oiled by oiling category and habitat type.

Lengths of shoreline by oiling category and State for three periods are summarized in Table 1 and Figure 2A: 1) Maximum oiling (highest degree of oiling ever observed on a shoreline); 2) Year 1 Post-Spill, (degree of oiling as of the most recent survey in the database on 1 May 2011); and 3) Year 2 Post-Spill, (oiling category as of the most recent survey in the database on 1 May 2012). Spatial extents of shoreline oiling categories for these same periods were also tracked (Figure 3). Figure 4 shows time-series plots of the lengths of shoreline by oiling category for the entire *Deepwater Horizon* spill response area for beaches and marshes. Tables S1, S2, and S3 in File S1 provide more detailed breakdowns by state and habitat for the three periods. For the maximum oiling table and map, “no oil observed” means that, based on the SCAT surveys, the shoreline was never oiled. For the Year 1 and Year 2 tables and maps, “no oil observed” means that, as of the last survey date within the period 1 May 2011 and 1 May 2012, the shoreline was not oiled. For these later periods, the shoreline might have been previously oiled, but that oil had been removed by cleanup actions and/or natural processes.

**Table 1:** Oiled shoreline lengths (km) by oiling category at maximum oiling conditions, one year (May 2011), and two years (May 2012) post spill^1^.

Length (km)	Total Surveyed	Heavy	Moderate	Light	Very Light	Trace (<1%)	Total Oiled	No Oil Observed
**Maximum Oiling**	7058	360	222	637	322	232	1,773	5,285
**One Year Post-Spill**	6967	22.4	56	178	131	459	847	6,120
**Two Years Post-Spill**	7057	6.4	17.5	91.6	83.7	488	687	6,370

Values rounded to nearest whole km. when greater than 100 km.

1 Shoreline oiling along the Texas coast was surveyed only once and using a slightly different approach, with a reported 58 km of trace oiling.

It is important to note that the most recent survey could have been conducted months prior to these reporting dates; however, all segments that had been documented as oiled were surveyed at least twice. The final survey for any given segment considered as operationally completed means that “no oil was observed” or that the oiling conditions met the appropriate guidelines or endpoints and did not require further cleanup treatment. (There was one important exception to this statement–the Chandeleur Islands in Louisiana, which are part of the Breton National Wildlife Refuge; SCAT teams were not able to conduct a final inspection before these segments were moved out of the response because Refuge staff had completed their own assessment and wanted to minimize any further disturbance to this highly sensitive and difficult to access location. Therefore, some of the persistent Heavy and Moderate oiling in Figure 4 represents the “oiling as of the last survey” on these islands and does not reflect actual conditions.) In some cases further cleanup was not conducted due to net environmental benefit considerations, where continued cleanup would either not improve or would worsen shoreline habitat conditions. In other locations, cleanup is ongoing (as of February 2013). Caution should be exercised in making specific comparisons of lengths reported here to shoreline lengths derived from other sources due to the fractal nature of shorelines and rapid shoreline change in the region, and potential differences in methods, criteria, or characteristics considered in studies of shoreline oiling for other purposes.

Of the 7,058 km of shoreline surveyed (Table 1), 1,773 km were documented as ever having been oiled (Heavy to Trace) across the entire affected area. The majority of these shorelines with documented oiling occurred in Louisiana (60.6%), followed by Florida (16.1%), Mississippi (14.6%), and Alabama (8.7%). For maximum oiling across all states, 20.3% of the shoreline oiling was classified as Heavy, 12.5% as Moderate, 35.9% as Light, 18.2% as Very Light, and 13.1% as Trace. Of the 1,773 km of shoreline that was ever observed as having been oiled, after one year 47.8% or 847 km still had some degree of oiling, and after two years, 38.8% or 687 km remained with some oil. In addition, heavy to moderately oiled shorelines had declined by 87% in one year and 96% in two years, compared to maximum oiling conditions.

Maximum shoreline oiling among major shoreline habitats (Figure 2B) was: beach (50.8% of the total; mostly sand beach but includes shell and mixed sand and shell beaches), marsh (44.9%; mostly coastal herbaceous marsh but includes mangroves and shell berms fronting marsh areas), and other (4.3%; mostly man-made shoreline types). Most of the marsh oiling (94.8%) occurred in Louisiana. Beach oiling was distributed throughout the four states with 32.9% in Louisiana, 31.3% in Florida, 21.1% in Mississippi, and 14.7% in Alabama.

SCAT teams evaluated the need for shoreline cleanup of all oiled shorelines and, where appropriate, recommended cleanup methods and constraints. Of the 900 km of beaches that were oiled, some type of shoreline treatment was conducted on 660 km, or 73.3% of oiled beaches. Many of the beaches affected were high-use, amenity beaches where the cleanup goals were “No visible oil above background levels or as low as reasonably practicable considering the allowed treatment methods and net environmental benefit,” thus extensive manual and mechanical cleanup operations were required. For non-amenity beaches and federally managed lands (national parks and wildlife refuges), the cleanup endpoint was <1% visible oil and other site-specific endpoints, thus less intensive cleanup was conducted to minimize ecological impacts. In contrast, of the 796 km of marshes that were oiled, shoreline treatment was allowed along 71 km, or 8.9% of oiled marshes and associated habitats (actual shoreline lengths treated were likely lower due to the patchy distribution of oil that required treatment in many marsh areas). Cleanup endpoints for marshes included no flushable oil, no release of sheens, and no thick (>1 cm) oil on the marsh platform. Much of the oil remaining two years after the spill was located in areas where additional cleanup or treatment would not provide a net environmental benefit or where the shoreline cleanup endpoints had been met. Thus, natural attenuation was often the recommended response action to avoid further damage to the marshes.

The trends in the degree of shoreline oiling over time on sand beaches and marshes (Figure 4) differed as a result of several factors: the intensive efforts to clean amenity beaches, chronic trace (<1% distribution) re-oiling on sand beaches along the eastern regions, the use of natural recovery for most of the marshes, senescence of oiled vegetation over the winter and emergence of new vegetation in spring (2011 and 2012), and the persistence of oil on the more sheltered marsh habitats.

## Discussion

Although every spill is a unique combination of conditions, the *Deepwater Horizon* spill response posed some particularly challenging shoreline oiling issues. The bulk of the oil stranded over more than a three-month period, and many of the Gulf of Mexico beaches were in an erosional state during the initial, heavy oiling, which led to burial of the oil as the beaches accreted over the following months. In addition, oil was stranded high in the supratidal due to high water levels and wave activity generated by storms in 2010. Over 180,000 pits, trenches, and augers holes were used to search for and delineate buried oil for removal through the end of 2012.

As the beaches went through the normal erosional and depositional processes of the beach cycle and seasonal wind patterns, the oil would become buried, exposed, and re-mobilized multiple times. Oil stranded on beaches in three zones. In the *supratidal zone* (from above normal tides to the toe of the dunes or end of overwash fans on beaches without dunes), oil was stranded in patches by storm waves. Wind patterns along the eastern Gulf of Mexico are such that winds are predominantly from the southeast from spring to fall [11], which deposited sand and buried oil residues in both the supratidal zone and as the beaches accreted. During the passage of winter cold fronts, strong winds blow from the north, which removed the sand via wind deflation, re-exposing some of the oil residues. Tropical Storm Lee (September 2011) and Hurricane Isaac August 2012) either eroded or buried more deeply the persistent oil residues in the supratidal zone, depending on location.

At locations in the *intertidal zone*, SRBs and SRPs became buried (>1 m in places). Tropical Storm Lee and Hurricane Isaac (the largest storms in the area between May 2010 and January 2013) caused extensive beach erosion and re-mobilization of oil residues, though some beaches had yet to fully erode back to their pre-spill profile. Removal of deeply buried oil residues has required extensive mechanical and manual excavation and sieving.

In the *lowest intertidal/nearshore subtidal zone*, there were two different patterns of oil accumulation. Along the more heavily oiled sand beaches in Florida, Alabama, and the offshore barrier islands of Mississippi, some of the oil/sand mixture accumulated in the nearshore subtidal, forming extensive submerged oil residue mats mostly between the toe of the beach and the first offshore bar. These mats were repeatedly buried and then exposed by sand migration. The subtidal oiled mats also became chronic sources of SRBs/SRPs on the adjacent shoreline as they broke up; in fact, the presence of angular SRBs/SRPs on the beach (Figure 1C) was a key indicator of the presence of subtidal nearshore oil residue mats.

Along the Louisiana barrier islands, oil/sand mixtures accumulated on portions of the lowermost intertidal zone, particularly where landward erosion of the barrier island exposed eroded relict marsh platforms composed of clay and peat at the toe of the sand beach. The oil/sand residues adhered to these surfaces, forming mats that were up to 100 m long and 20 cm thick. These mats were only exposed during the lowest of tides and/or were buried by beach accretion, making it difficult to delineate and remove them. These mats were also chronic sources of SRBs/SRPs on the adjacent beaches, as described above.

Along most of the marshes, the oil stranded along the marsh edge and bulk oiling usually spread into the marsh no more than about 10–15 m perpendicular to the shoreline due to the small tidal range (∼0.5 m), the density of the vegetation, and the residual oil’s high viscosity. The heaviest marsh oiling was most widespread in salt marshes (*Spartina alterniflora*, *Juncus roemerianus*) in northern Barataria Bay, Louisiana [12] [13] [14] [15]. Other marsh locations that required treatment, but over smaller areas, were documented in Terrebonne-Timbalier Bays (e.g., Casse Tete Island) and the outer islands of Biloxi Marsh (e.g., Keel Boat Pass Island), as well as in Roseau cane marshes (*Phragmites australis*) on the Mississippi Delta (e.g., Pass a Loutre). Heavy persistent oiling conditions in northern Barataria Bay and other salt marshes included heavily oiled vegetation mats (above-ground vegetation laid over by oiling, which died but remained rooted in place) and wrack lines that in many cases overlaid a thick layer of emulsified oil on the marsh substrate. In the fall of 2010, much of the heavily oiled layer on marsh platforms averaged 2–3 cm in thickness and did not appear to have significantly weathered or naturally degraded [12]. Over 11 km of the most heavily oiled marshes in northern Barataria Bay were cleaned using intensive manual and mechanical raking and cutting methods to remove the oiled vegetation mats and wrack, careful removal or reduction of the thick oil layers on the substrate, and limited application of loose organic sorbents [12], [13].

Not every spill response includes such a comprehensive SCAT program, though some sort of shoreline surveys are always conducted to determine where response is needed. To put the *Deepwater Horizon* SCAT results in perspective, Table 2 presents shoreline oiling data from two other major oil spills–the *Exxon Valdez* in Alaska and the Gulf War oil spill in the Arabian Gulf, along with two smaller spills with detailed SCAT data. Obviously, there is little relationship between spill volume and length of shoreline oiled. The oil from the *Exxon Valdez* was transported over long distances by the Alaska Coastal Current; in contrast, the oil from the Gulf War oil spill mostly hugged the shoreline because of unusual northerly winds, so little oil got beyond the headlands formed by Abu Ali Island near Jubail, Saudi Arabia.

**Table 2:** Comparison of the lengths of shoreline oiled for systematic surveys.

Spill Name/Date	Oil Type/Volume	Shoreline Area Oiled	ShorelineSurveyed (km)	ShorelineOiled (km)
T/V *Exxon Valdez* March1989 [16]	Alaska North Slope crudeoil/260,000 barrels	Prince William Sound, Kenai Peninsula,and Kodiak Strait, Alaska	5,459	2,100
Gulf War oil spill February-May1991 [17]	Kuwait crude oil/10,800,000barrels	Saudi Arabia shoreline of the westernArabian Gulf (limited but unknownarea oiled in Kuwait)	772	707
T/V *Selendang Ayu* December2004 [18]	Intermediate fuel oil 180+ marine diesel/ 8,434 barrels	Western shoreline of Unalaska Island, Alaska	763	418
M/V *Cosco Busan* November2007 [19]	Intermediate fuel oil 380/1,380barrels	Central San Francisco Bay and outershorelines north and south of theGolden Gate, California	379	147
*Deepwater Horizon*,April-August 2010	MC-252 Louisiana crudeoil/4,900,000 barrels	Northeastern Gulf of Mexico	7,057	1,773

There are other differences among the data in Table 2. The oiled band width defined as Heavy for the *Exxon Valdez* response surveys is >6 m, whereas for the *Deepwater Horizon* response it is >1.8 m, reflecting the differences in tidal range among the regions (5 m in Alaska and <1 m in the Gulf of Mexico). Along the Arabian Gulf, the width of the oiled band was often in the tens of meters and exceeded 1–2 km on the extensive intertidal flats with mostly 100% oil cover and deep penetration into the sediments [17] [20].

As is the case for any field data-collection project, SCAT requires adherence to standard methods of field observation and measurements by calibrated field teams. Consistency among teams over time is essential and a deliberate effort was made to maintain the same cadre of team leaders throughout the response, with frequent calibration as oiling conditions changed. The field data went through rigorous automated and visual checks to assure data quality; a large number of stakeholders relied on the quality and objectiveness of the field data to support decision making at all levels of the response. SCAT during the *Deepwater Horizon* spill was not different from surveys conducted on other spills, except in the scope and duration.

The SCAT Program for the *Deepwater Horizon* oil spill response was understandably large, complex, and involved many stakeholders across four states and multiple jurisdictions. The traditional SCAT model was modified to fit the environmental, operational, and political challenges posed by the scale of the incident and information demands of the Unified Command [21]. As of early 2013, the SCAT Program continues to generate data to support cleanup decision-making, track oiling conditions over time, track cleanup progress and efficacy, and ensure shorelines meet endpoints.

## Supporting Information

File S1 Figure S1, The two step process by which the shoreline oiling descriptors generate the oiling degree category to be assigned to each shoreline segment. In the first step, the width of the oiled band and the % oil distribution determine the initial oiling category; in the second step, the oil thickness determines the final oiling category. Table S1, Detailed breakdown of the kilometers of shoreline oiled by State, habitat, and oiling degree for the maximum oiling. Table S2, Detailed breakdown of the kilometers of shoreline oiled by State and oiling degree at 1 year post-release. Table S3, Detailed breakdown of the kilometers of shoreline oiled by State and oiling degree at 2 years post-release.
(DOCX)
