# Supplementary material for: Extent and Degree of Shoreline Oiling: Deepwater Horizon Oil Spill, Gulf of Mexico, USA
Source: PLoS One. 2013 Jun 12;8(6):e65087. doi: 10.1371/journal.pone.0065087 (PMC3680451; doi:10.1371/journal.pone.0065087)
Supplement: File S1 — Figure S1, The two step process by which the shoreline oiling descriptors generate the oiling degree category to be assigned to each shoreline segment. In the first step, the width of the oiled band and the % oil distribution determine the initial oiling category; in the second step, the oil thickness determines the final oiling category. Table S1, Detailed breakdown of the kilometers of shoreline oiled by State, habitat, and oiling degree for the maximum oiling. Table S2, Detailed breakdown of the kilometers of shoreline oiled by State and oiling degree at 1 year post-release. Table S3, Detailed breakdown of the kilometers of shoreline oiled by State and oiling degree at 2 years post-release. (DOCX) [file pone.0065087.s001.docx]

**Supplementary Materials:**

Step 1 in defining oiling degree

|  | **Shore-Perpendicular Oiled Band Width** | | | |
| --- | --- | --- | --- | --- |
| **Oil Dist.** | **Wide**  **>1.8m** | **Medium**  **0.9-1.8m** | **Narrow**  **0.3-0.9m** | **Very Narrow**  **<0.3m** |
| **Continuous**  **>90%** | Heavy | Heavy | Moderate | Light |
| **Broken**  **51-90%** | Heavy | Heavy | Moderate | Light |
| **Patchy**  **11-50%** | Moderate | Moderate | Light | Very Light |
| **Sporadic**  **1-10%** | Light | Light | Very Light | Very Light |
| **Trace**  **<1%** | Trace | Trace | Trace | Trace |

Step 2 in defining oiling degree

|  | **Initial Categorization** | | | |
| --- | --- | --- | --- | --- |
| **Average Oil Thickness** | **Heavy** | **Moderate** | **Light** | **Very Light** |
| **Thick**  **>1 cm** | Heavy | Heavy | Moderate | Light |
| **Cover**  **0.1-1.0 cm** | Heavy | Heavy | Light | Light |
| **Coat**  **0.01-0.1 cm** | Moderate | Moderate | Light | Very Light |
| **Stain/Film**  **<0.01 cm** | Light | Light | Very Light | Very Light |

Figure S1. The two step process by which the shoreline oiling descriptors generate the oiling degree category to be assigned to each shoreline segment. In the first step, the width of the oiled band and the % oil distribution determine the initial oiling category; in the second step, the oil thickness determines the final oiling category.

Table S1. Detailed breakdown of the kilometers of shoreline oiled by State, habitat, and oiling degree for the maximum oiling^1^.

| **DETAILED BREAKDOWN OF MAXIMUM OILING by HABITAT** | | | | | | | | |
| --- | --- | --- | --- | --- | --- | --- | --- | --- |
| **State/ Shoreline Habitat** | **Total Surveyed** | **Heavy** | **Moderate** | **Light** | **Very Light** | **Trace (<1%)** | **Total Oiled** | **No Oil Observed** |
| **Alabama** |  |  |  |  |  |  |  |  |
| Beach | 141.8 | 61.7 | 0.7 | 46.6 | 0.0 | 23.2 | 132.3 | 9.5 |
| Marsh | 101.6 | 0.0 | 0.4 | 4.0 | 1.4 | 4.4 | 10.1 | 91.5 |
| Other | 198.1 | 0.5 | 0.6 | 8.4 | 1.4 | 1.2 | 12.1 | 186.0 |
| **AL - Totals** | **441.5** | **62.2** | **1.7** | **59.0** | **2.7** | **28.9** | **154.5** | **287.0** |
|  | | | | | | | | |
| **Florida** |  |  |  |  |  |  |  |  |
| Beach | 615.4 | 49.3 | 0.0 | 121.0 | 5.6 | 106.4 | 282.2 | 333.2 |
| Marsh | 138.3 | 0.0 | 0.0 | 0.0 | 0.0 | 0.0 | 0.0 | 138.3 |
| Other | 87.6 | 0.1 | 0.0 | 0.8 | 1.0 | 1.5 | 3.4 | 84.2 |
| **FL Totals** | **841.3** | **49.4** | **0.0** | **121.8** | **6.5** | **107.9** | **285.6** | **555.7** |
|  | | | | | | | | |
| **Louisiana** |  |  |  |  |  |  |  |  |
| Beach | 425.2 | 86.4 | 33.2 | 90.9 | 42.9 | 42.8 | 296.2 | 129.1 |
| Marsh | 4697.7 | 134.7 | 169.1 | 202.8 | 222.8 | 24.9 | 754.2 | 3943.5 |
| Other | 174.6 | 8.6 | 4.5 | 3.0 | 5.8 | 2.2 | 24.1 | 150.5 |
| **LA Totals** | **5297.5** | **229.7** | **206.8** | **296.7** | **271.5** | **69.9** | **1074.5** | **4223.0** |
|  |  |  |  |  |  |  |  |  |
| **Mississippi** |  |  |  |  |  |  |  |  |
| Beach | 220.2 | 17.8 | 9.4 | 127.9 | 15.2 | 19.3 | 189.6 | 30.6 |
| Marsh | 129.9 | 0.1 | 1.5 | 20.4 | 8.5 | 1.0 | 31.5 | 98.3 |
| Other | 127.2 | 0.5 | 2.9 | 11.1 | 17.7 | 5.1 | 37.3 | 89.9 |
| **MS Totals** | **477.3** | **18.4** | **13.9** | **159.4** | **41.4** | **25.4** | **258.5** | **218.8** |
|  |  |  |  |  |  |  |  |  |
| **All Totals** |  | | | | | | | |
| Beach | 1402.7 | 215.3 | 43.3 | 386.3 | 63.7 | 191.7 | 900.3 | 502.4 |
| Marsh | 5067.5 | 134.8 | 171.0 | 227.2 | 232.6 | 30.3 | 795.9 | 4271.6 |
| Other | 587.5 | 9.7 | 8.0 | 23.4 | 25.8 | 10.1 | 76.9 | 510.5 |
| **All Totals** | **7057.7** | **359.8** | **222.3** | **636.8** | **322.1** | **232.1** | **1773.1** | **5284.5** |

^1^ Based on data as of 1 May 2012

Table S2. Detailed breakdown of the kilometers of shoreline oiled by State and oiling degree at 1 year post-release^1^.

| **DETAILED BREAKDOWN OF SURFACE OILING by STATE, 1 Year Post-Release** | | | | | | | | |
| --- | --- | --- | --- | --- | --- | --- | --- | --- |
| **Shoreline Habitat** | **Total Surveyed** | **Heavy** | **Moderate** | **Light** | **Very Light** | **Trace (<1%)** | **Total Oiled** | **No Oil Observed** |
| Alabama | 441.8 | 0.0 | 0.3 | 12.0 | 1.9 | 77.2 | 91.4 | 350.4 |
| Florida | 841.1 | 0.0 | 0.0 | 0.1 | 0.3 | 140.1 | 140.5 | 700.6 |
| Louisiana | 5207.0 | 22.4 | 55.3 | 142.5 | 124.0 | 120.9 | 465.1 | 4741.9 |
| Mississippi | 477.3 | 0.0 | 0.3 | 23.5 | 4.8 | 121.1 | 149.8 | 327.5 |
| **Totals** | **6967.2** | **22.4** | **56.0** | **178.0** | **131.0** | **459.3** | **846.8** | **6120.3** |

^1^ Based on data as of 1 May 2012

Table S3. Detailed breakdown of the kilometers of shoreline oiled by State and oiling degree at 2 years post-release^1^.

| **DETAILED BREAKDOWN OF SURFACE OILING by STATE, 2 Years Post-Release** | | | | | | | | |
| --- | --- | --- | --- | --- | --- | --- | --- | --- |
| **Shoreline Habitat** | **Total Surveyed** | **Heavy** | **Moderate** | **Light** | **Very Light** | **Trace (<1%)** | **Total Oiled** | **No Oil Observed** |
| Alabama | 441.5 | 0.0 | 0.0 | 0.9 | 0.0 | 94.1 | 95.0 | 346.5 |
| Florida | 841.3 | 0.0 | 0.0 | 0.0 | 0.0 | 115.1 | 115.1 | 726.2 |
| Louisiana | 5296.7 | 6.4 | 15.9 | 81.7 | 82.7 | 160.4 | 347.2 | 4949.5 |
| Mississippi | 477.3 | 0.0 | 1.6 | 8.9 | 0.9 | 118.5 | 130.0 | 347.4 |
| **Totals** | **7056.9** | **6.4** | **17.5** | **91.6** | **83.7** | **488.0** | **687.2** | **6369.6** |

^1^ Based on data as of 1 May 2012
